# Accuracy of Various Iron Parameters in the Prediction of Iron Deficiency Anemia among Healthy Women of Child Bearing Age, Saudi Arabia

**Published:** 2012-07-30

**Authors:** J M AlQuaiz, H M Abdulghani, R A Khawaja, S Shaffi-Ahamed

**Affiliations:** 1 Department of Family and Community Medicine, King Khalid University Hospital, King Saud University, College of Medicine, Riyadh, Saudi Arabia; 2 Department of Medical Education, King Khalid University Hospital, King Saud University, College of Medicine, Riyadh, Saudi Arabia; 3 Department of Family Medicine, King Khalid University Hospital, King Saud University, College of Medicine, Riyadh, Saudi Arabia; 4 Chai of Princess Nora for Women’s Health Research, King Khalid University Hospital, King Saud University, College of Medicine, Riyadh, Saudi Arabia

**Keywords:** Iron deficiency anemia, Women, Ferritin, MCV, MCH, RDW

## Abstract

**Background:**

Iron deficiency is still considered the most common nutritional deficiency worldwide and the most significant negative consequence of iron deficiency is iron deficiency anemia (IDA). This study elucidates if IDA among healthy women of child bearing age could be predicted by various iron parameters, using serum ferritin as a gold standard.

**Methods:**

Between January and June 2009 at primary care clinics of a teaching hospital in Saudi Arabia, 112 anemic (Hemoglobin = 120 g/l) subjects participated in the study. Mean corpuscular volume (MCV), mean corpuscular hemoglobin (MCH), mean corpuscular hemoglobin concentration (MCHC), red blood cell distribution width (RDW), serum ferritin, and hemoglobin electrophoresis were obtained from all participants. Receiver operator characteristic (ROC) curves were used to assess the accuracy of various iron parameters.

**Results:**

With respect to the serum ferritin, the best predictive cut-off value of MCV, MCH and RDW at the most optimal were 76 fl (ROC curve=0.768), 24 Pg (ROC curve=0.72) and 16.1% (ROC curve=0.711), respectively. MCHC was not significant in predicting the iron deficiency in these patients.

**Conclusion:**

IDA can be predicted among women of child bearing age using complete blood count test. MCV, MCH and RDW are the iron parameters of complete blood count test, which is a cost effective, easily accessible and could be useful tool in areas with limited resources and a high prevalence of the disease.

## Introduction

Iron deficiency is still considered the most common nutritional deficiency worldwide and the most significant negative consequence of iron deficiency is iron deficiency anemia (IDA).[1][2] It can decrease the ability to work, causing tiredness and chronic fatigue in adults,[3][4][5] affecting cognitive function in adolescent girls and resulting in negative impact on motor and mental development in children and adolescents. It may also affect visual and auditory function of affected person.[5]

Prevalence of IDA varies widely by age, sex, race and geographical areas.[6][7] Studies have shown the prevalence of 15-36% in elderly,[8][9] with relatively more prevalence in female population.[10] Among women of child bearing age, the prevalence of anemia in Indonesia was about 30-40%.[11] Another study from Indonesia revealed higher prevalence of anemia among women (15.9%) as compared to men (3.9%).[12]

A study in Saudi Arabia reported prevalence of IDA by 20.5%, among school students. Anemia in that study was more prevalent among students of ≥12 years of age as compared to the younger age group and was associated with negative impact on school performance and was more marked among those who failed their exams as compared to students with excellent results.[13] More than one fourth of girls in Saudi Arabia have serum iron below 100 g/L and the most affected ones are in the age group of 7-14 years.[14] In another study, IDA was relatively more common among menstruating women and girls and were twice more likely to be anemic than non-menstruating girls.[15]

The classical and most commonly used hematological screening test for iron deficiency is hemoglobin estimation.[12] Relying on hemoglobin alone for screening delays the detection of IDA because, sufficient time must elapse for iron to have an impact.[16] Hence, there is a need to have a screening test which is cheap and of high reliability and accuracy in identifying the iron deficiency. The definitive test for the diagnosis of iron deficiency anemia is the bone marrow aspiration (to assess the iron stores). The procedure, however, is invasive, difficult and expensive for a very common medical problem especially in developing countries. Alternate to bone marrow aspiration test is serum ferritin, which is found to be the best test for distinguishing those with IDA from those who are not iron deficient.[8][17][18][19][20][21][22] Unfortunately, serum ferritin estimation test is not available freely at many primary health care centers, especially in developing countries. Therefore, this study was conducted to investigate if anemia among healthy women of child bearing age caused by iron deficiency could be detected by simple parameters obtained on a complete blood count, using serum ferritin level as a standard of reference.

## Materials and Methods

This is a pilot study conducted between January and June 2009, at seven primary care clinics (serving only adult female patients) at King Khalid University Hospital (KKUH), Riyadh, Saudi Arabia. Approval from Medical College Research Committee' (MCRC) was obtained. Additional patients' consent form was filled by the participants.

We recruited Saudi females of child bearing age (15 to 49 years, as defined by WHO)[23] who visited primary care clinics during the study period and had a hemoglobin level <120 g/l and who were willing to participate in the study. We excluded those who were post-menopausal, pregnant, lactating mothers, with pregnancy related conditions for the last three months (abortion and post-partum period), post-hysterectomy, on treatment with iron tablets at any time during the last 6 months, known case of chronic diseases including patients on renal dialysis and having any type of hemoglobinopathy or acute and chronic infection or inflammation.

Laboratory investigations carried out on each subject were complete blood count (CBC), serum ferritin and haemoglobin electrophoresis. One hematologist was assigned to follow, analyze and complete the hematological data.

Anemia was defined as hemoglobin <120 g/l (cut off point according to WHO criteria).[21] Microcytic anemia was defined as anemia with MCV (mean cell volume) less than 80 fL (normal 80-96 fl). IDA was defined as anemia with ferritin level ≤ 15 ng/ml.[20][21][24] Serum ferritin was measured fasting using a microparticle enzyme immunoassay on the Abbott AxSym chemistry analyzer (Abbott Laboratories, Abbott Park, Illinois, USA). Red cell distribution width (RDW) was calculated as (standard deviation of MCV/mean MCV) x100 and was reported as a percentage (Normal value is 11% - 14.5%).[20]

The data was processed in a microcomputer and the statistical software (SPSS, Version 16.0, Chicago, Il, USA) was used for statistical analysis. The accuracy of the various iron parameters was analyzed using MedCalcTM statistical software program (MedCalc Software, Belgium) and plotting receiver operating characteristic (ROC) curves. We assessed the accuracy of various iron parameters in the prediction of iron deficiency, using the Hb (Below 120 g/l) and serum ferritin (≤ 15 ng/ml) as a gold standard for diagnosing the iron deficiency anemia.

The diagnostic performances (sensitivity, specificity, positive and negative likelihood ratios) of the various iron parameters at different cut-off points for the diagnosis of iron deficiency were plotted on a ROC curve. From the ROC curves, the levels of the various iron parameters at their optimal likelihood ratios for diagnosing iron deficiency were derived.

## Results

Out of 135 participants who were enrolled in the pilot study, only 112 women satisfied the recruit meat catena. Twenty three women were excluded, 2 had thalasemia trait, one had sickle cell trait, 10 participants did not complete their investigations and 10 had either chronic renal failure (2) or acute infections (8) on the basis of investigations.

Table 1 shows the socio-demographic characteristics of anemic women. Twenty nine percent of women in the study group were in the age group of 30-39 years and 27% in the age group < 20 years. More than the half of the study population were housewives and/or married. A quarter of the study population was illiterate and about a quarter of spouses or fathers were skilled workers. About one third of population lived either in flats or small houses. The majority of study populations (82%) were from urban areas. The majority of patients (64%) had mild anemia with hemoglobin level of ≥ 100 – <120 g/l, 31% had moderate (≥ 80- <100 g/l) and only about 5% had severe anemia (<80 g/l). Fifty four participants’ serum ferritin was <15 ng/ml and counted as an abnormal test.

**Table 1:** Socio-demographic characteristics of women of child bearing age with iron deficiency anemia.

**Socio-demographic characteristics**	**Cases (112) No. (%)**
Age	
<20	30 (26.8)
20 – 29	29 (25.9)
30 – 39	32 (28.6)
≥40	21 (18.7)
Marital status	
Single	40 (35.7)
Married	63 (56.3)
Divorced/widow	9 (8.0)
Education	
Illiterate	27 (24.1)
Elementary	21 (18.8)
Intermediate	23 (20.5)
Secondary	25 (22.3)
University	16 (14.3)
Occupation	
Housewife	65 (58.0)
School teacher	5 (4.5)
Students	28 (25.0)
Other	14 (12.5)
Spouse or Guardian‘s occupation	
High professional	22 (19.6)
Middle professional	28 (25.0)
Skilled/M.	30 (26.8)
Unskilled	16 (14.3)
Others	16 (14.3)
Geographical Location	
Urban	92 (82.1)
Suburban	20 (17.9)
Housing	
Villa	76 (67.9)
Flat	19 (17.0)
Small house	17 (15.1)
Smoking	
Yes	4 (3.6)
No	108 (96.4)

**Fig. 1:**
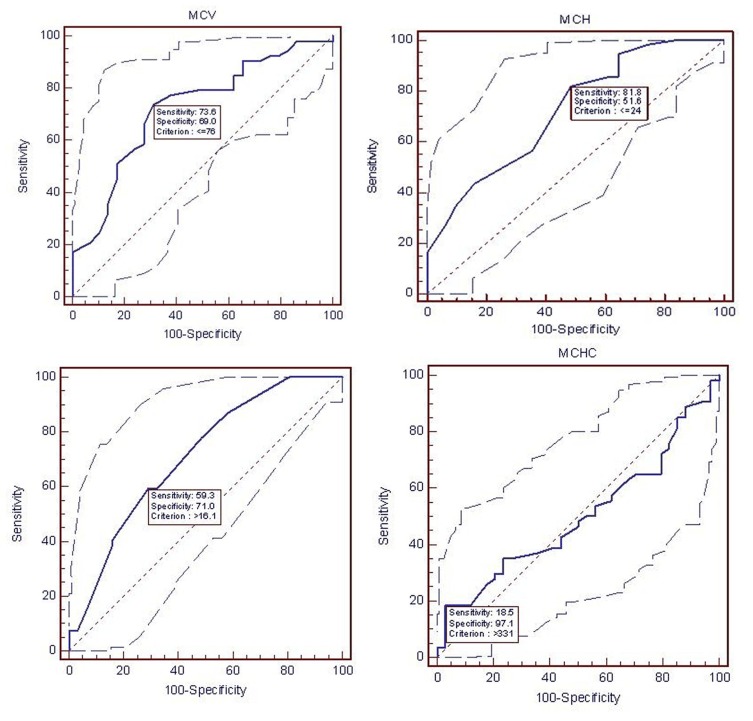
(A) Area under the ROC curve (AUC)=0.724 (95 % CI=0.614 – 0.817, p-value=0.0003 and +LR 2.37), (B) Area under the ROC curve (AUC)=0.721 (95 % CI=0.614 – 0.812, p-value=0.0002 and +LR 1.69), (C) Area under the ROC curve (AUC)=0.711 (95 % CI=0.602 – 0.804, p-value=0.0002 and +LR 2.04), (D) Area under the ROC curve (AUC)=0.500 (95 % CI=0.391 – 0.609, p-value=1.0 and +LR 6.3).

The ROC curve for iron parameters i.e. MCV, MCH, RDW and MCHC, together with the area under the curves with their 95% CI and p value were shown in Figure 1. Among healthy women of child bearing age, the best marker in comparison with serum ferritin for predicting the presence of iron deficiency was MCV followed by MCH and then RDW. MCHC was not significant in predicting the iron deficiency in this group of participants.

From the ROC curves, the level of MCV, MCH, and RWD at the optimal positive likelihood ratio for diagnosis of iron deficiency (likelihood ratio=2.37, 1.69, 2.04) was found to be 76 fl, 24 Pg and 16.1% respectively.

## Discussion

The purpose of this study was to characterize the performance of simple, easily accessible, cost effective and commonly used diagnostic test for iron deficiency anemia in developing countries and especially at primary care level. The serum ferritin assay has been considered the best single test for the diagnosis of iron deficiency anemia. Serum ferritin at a very low level (<14 ng/ml) gives very high likelihood ratio (LR+42)[8] and is the most powerful test with an area under the ROC curve of 0.95.[18] Hence, we used serum ferritin as a gold standard for the diagnosis of IDA in this study and compared it to other iron parameters.

In a review of 55 studies, MCV at the level of <70 fl had a good predictive ability for diagnosing IDA with LR+12.[18] In contrast to the review, our study indicated that 76 fl was the appropriate cutoff value of MCV for the diagnosis of iron deficiency and at the level of 76 fl, MCV had a sensitivity of 73.6% and specificity 69% with LR+2.37. This slight difference of MCV level for the diagnosis of IDA could be due to the gold standard for comparison, which is bone marrow in a review study as compared to serum ferritin in our study.

In comparison to our results, many studies have found that RDW, MCV, and MCH are important parameters for screening and detecting IDA.[16][18][22][25][26] Among the different laboratory test results, sensitivity of RDW was the highest (89%) and LR+2.6, followed by MCH and MCV (84%) compared to only 63% in MCHC. Sensitivity of RDW, MCV and MCH was quite lower (59.3, 73.6 and 81.8%, respectively) in this study. This could be explained by smaller sample size and different level of criterion. Furthermore, care to be taken regarding the time to see changes in these parameters. Earliest change will take place in MCV (21 days) followed by RDW (30 days) and Hb may take up to 60 days to become low.[20]

A study among pregnant females of <20 wks gestation showed that a RDW ≥ 15 and a Hb <9.7 mg/dL correctly diagnosed IDA (ROC 0.9, with sensitivity and specificity of 46.8% and 95.7%, respectively). RDW ≥ 15 in that study proved to be a highly specific predictor of IDA in pregnancy.[27] Whereas, in our study of non-pregnant but child bearing age females showed RDW of ≥ 16.1% to diagnose IDA with reasonable accuracy (Area under the ROC curve=0.711, sensitivity of 59.3%, specificity of 71% and +LR 2.04).

Uniformity of the study population is an obvious strength of this study. Whereas, the main study limitation includes relatively small number of patients which limits the confidence with which one can generalize the results. The relatively small number of patients reduces the precisions of the likelihood ratios as well. In the presence of these limitations, the study has revealed some important findings.

In conclusion, our data showed that CBC indices were the good alternate predictor of iron deficiency in women of child bearing age. Diagnosing iron deficiency based on CBC is simple, inexpensive, rapid and practical to perform. We supposed that CBC is an ideal test to early predict iron deficiency. Further studies are needed to determine whether CBC should be the preferred screening tool in the early detection of iron deficiency in larger, unselected population of both sexes and in all age groups.

Caution is urged in extrapolating the results of this study in mostly women of child bearing age to other patient populations. Furthermore, when there is any clinical ambiguity, the clinician may still need to do serum ferritin level and/or examine bone marrow for iron stores to diagnose iron deficiency anemia.
